# Immunological aspects of host–pathogen crosstalk in the co-pathogenesis of diabetes and latent tuberculosis

**DOI:** 10.3389/fcimb.2022.957512

**Published:** 2023-01-26

**Authors:** Arpana Verma, Maninder Kaur, Princy Luthra, Lakshyaveer Singh, Divya Aggarwal, Indu Verma, Bishan D. Radotra, Sanjay Kumar Bhadada, Sadhna Sharma

**Affiliations:** ^1^ Department of Biochemistry, Post Graduate Institute of Medical Education and Research, Chandigarh, India; ^2^ Tuberculosis Aerosol Challenge Facility (TACF), International Centre for Genetic Engineering and Biotechnology, New Delhi, India; ^3^ Department of Histopathology, Post Graduate Institute of Medical Education and Research, Chandigarh, India; ^4^ Department of Endocrinology, Post Graduate Institute of Medical Education and Research, Chandigarh, India

**Keywords:** Latent tuberculosis, diabetes, triglyceride synthase, matrix metalloproteinases, cytokines

## Abstract

**Introduction:**

Diabetes is a potent risk factor for the activation of latent tuberculosis and worsens the tuberculosis (TB) treatment outcome. The major reason for mortality and morbidity in diabetic patients is due to their increased susceptibility to TB. Thus, the study was conducted to understand the crosstalk between *M. tuberculosis* and its host upon latent tuberculosis infection and under hyperglycemic conditions or diabetes.

**Methods:**

An animal model was employed to study the relationship between latent tuberculosis and diabetes. BCG immunization was done in mice before infection with *M. tuberculosis*, and latency was confirmed by bacillary load, histopathological changes in the lungs and gene expression of *hspX, tgs1, tgs3* and *tgs5*. Diabetes was then induced by a single high dose of streptozotocin (150 mg/kg body weight). Host factors, like various cytokines and MMPs (Matrix metalloproteinases), which play an important role in the containment of mycobacterial infection were studied *in vivo* and *in vitro*.

**Results:**

A murine model of latent TB was developed, which was confirmed by CFU counts (<10^4^ in the lungs and spleen) and granuloma formation in lungs in the latent TB group. Also, the gene expression of *hspX, tgs1*, and *tgs5* was upregulated, and after diabetes induction, blood glucose levels were >200 mg/dl. An *in vitro* study employing a THP-1 macrophage model of latent and active tuberculosis under normal and high glucose conditions showed that dormant bacilli were better contained in the presence of 5.5 mM glucose concentration as compared with active bacilli. However, the killing and restriction efficiency of macrophages decreased, and CFU counts increased significantly with an increase in glucose concentration.

**Discussion:**

The decreased levels of MCP-1, decreased expression of *mmp-9*, and increased expression of *mmp-1* in the latent group at high glucose concentrations could explain the failure of granuloma formation at high glucose conditions.

## 1 Introduction

Diabetic patients are twice as vulnerable to *M. tuberculosis* infection compared to non-diabetics, and diabetes also increases the risk of premature death in tuberculosis patients (Faurholt-Jepsen et al., 2013). There is a two-way association between tuberculosis and diabetes. Although diabetes is a potent risk factor for TB and affects treatment outcomes (Baker et al., 2011), tuberculosis, on the other hand, influences glycemic control in diabetic patients as well (Fisher-Hoch et al., 2013). At present, the mechanisms behind the association between tuberculosis and diabetes are not completely known. Compromised immunological condition plays a crucial role in the susceptibility to the tuberculosis. As diabetes is a pandemic, it is now among the well-known causes for an immune compromised state, which facilitates TB development along with HIV, aging, malnutrition, and smoking (Fox and Menzies, 2013; Rieder, 2014). In diabetes, the immunity of the host against *M. tuberculosis* is “dysfunctional” to be precise instead of “compromised” as in the case of other risk factors with exaggerated or delayed immune responses against *M. tuberculosis*, which renders the host susceptible for activation of latent tuberculosis.

Monocytes play a crucial role in tuberculosis by migrating to the lungs in response to *M. tuberculosis* exposure and differentiating into macrophages and dendritic cells that act as antigen-presenting cells and release cytokines. Chronic hyperglycemia alters the expression of various chemokines and cytokines, causes phagocytic dysfunction in monocytes, and inhibits the complement system, thereby affecting the immune system directly (Stegenga et al., 2008; Hair et al., 2012; Restrepo et al., 2014). The immune susceptibility of diabetics to TB is not clearly understood. Increased TB susceptibility in diabetes patients can be attributed to multiple factors, with direct effects linked to insulin resistance and hyperglycemia, whereas indirect effects have been linked to lymphocyte and macrophage function (Dooley and Chaisson, 2009; Restrepo and Schlesinger, 2013; Martinez and Kornfeld, 2014). Compromised immunity in diabetic patients, which either reactivates latent tuberculosis or promotes primary infection of tuberculosis, could be a possible explanation for overall impaired immunity (Ponce-de-Leon et al., 2004).

MMPs are tightly regulated by various growth factors, hormones, cytokines and are also controlled by tissue inhibitors of MMPs (TIMPs) and endogenous MMP inhibitors (MMPIs). Cytokines modulate MMPs production at the gene level *via* negative or positive regulatory elements (Woessner, 1991), and may affect the proteolytic enzymes production that inhibit or activate MMPs (Ries and Petrides, 1995; Galboiz et al., 2002). Thus, during *M. tuberculosis* infection, where cytokine levels are upscaled, the activity and regulation of MMPs is expected to be controlled by cytokines. MMPs intercede in the immunological response toward infectious pathogens (Turner et al., 2001; Lee et al., 2005). MMPs, particularly MMP-9, have been reported to be expressed during various types of tuberculosis, including meningitis (Matsuura et al., 2000; Price et al., 2001; Thwaites et al., 2003; Lee et al., 2004), active cavitary tuberculosis (Chang et al., 1996; Price et al., 2001; Hrabec et al., 2002), and pleuritis (Hoheisel et al., 2001; Hrabec et al., 2002; Jin et al., 2004). Inhibition of MMP-9 along with the TB treatment has shown increased bacillary clearance and inhibited inflammation in tuberculosis meningitis (Majeed et al., 2016). Thus, the emerging data suggest an important role of MMPs in tuberculosis and its various associated pathological states. Extracellular matrix (ECM) plays a vital role in the structural composition of granuloma in terms of leucocyte trafficking in and out of this dynamic environment (Rhoades, 1997; Gonzalez-Juarrero et al., 2001), but the underlying events during this structural reorganization to establish a stable granuloma or advancement toward a pathological lesion as the granuloma dissolves are unfolding slowly. Interestingly, MMPs are the key factors involved in both the granuloma creation and lung tissue destruction (Salgame, 2011). So, the present study was focused on understanding the crosstalk between *M. tuberculosis* and host upon latent TB infection under hyperglycemic conditions/diabetes with respect to various cytokines and MMPs involved in granuloma formation in TB diabetes copathogenesis.

## 2 Materials and methods

### 2.1 Animal procurement and ethics statement

All animal procedures were performed in the Bio-Safety Level-III (BSL-III) facility at the International Centre for Genetic Engineering and Biotechnology (ICGEB), New Delhi. Animal procedures were approved by the Institutional Animal Ethical Committee with ref. no. 89/90/IAEC/616 and also by the Animal Ethical Committee of the ICGEB, New Delhi, with ref. no. ICGEB/IAEC/02042019/TACF-PGIMER-16 (Verma et al., 2021).

### 2.2 *M. tuberculosis* H_37_Rv culture and maintenance

*M. tuberculosis* H_37_Rv (NCTC-7416), originally procured from the national collection of type culture in London, UK, was cultured in Soutan’s media containing 2% glycerol and 0.05% Tween 80 under shaking conditions at 200 rpm. Logarithmically growing cultures were enumerated by comparing with McFarland standards and stored at −80°C till further use.

### 2.3 Mouse model of latent tuberculosis and diabetes

The mouse model of latent tuberculosis was developed by infecting 5 to 6-week-old Balb/c mice with BCG through aerosolization at 0.5 O.D. (600 nm), and after six weeks, *M. tuberculosis* H37Rv infection was given at 1.1 O.D. (600 nm). Latency was confirmed by CFU counts after 4 and 6 weeks of infection with *M. tuberculosis*. Six weeks post infection with *M. tuberculosis*, latently infected animals were divided into three groups, i.e., Group-I: latent tuberculosis only (n = 24), Group-II: latent tuberculosis with diabetes (n = 24), and Group-III: latent tuberculosis with immunosuppression (n = 24). Group-I animals were kept untreated. Group-II animals were induced with diabetes by a single dose of streptozotocin (150 mg/kg body weight), and Group-III animals were induced with immunosuppression by dexamethasone (Ahmad et al., 2006). Animals were sacrificed at weeks 9 and 13 of diabetes induction.

### 2.4 Histopathological studies

Aseptically removed lungs and spleen were transferred to 10% buffered formalin and further processed for paraffin embedding and sectioning (Ramos-Vara, 2011). Paraffin sections were deparaffinized and stained with the standard hematoxylin and eosin (H&E) and the acid-fast bacilli (AFB) stain.

### 2.5 Culture and maintenance of THP-1 cell line

The human leukemic monocytic cell line THP-1 was obtained from the cell repository at the National Centre for Cell Science (NCCS) Pune, India, and was routinely maintained as suspended cells in RPMI 1640 media, supplemented with 10% fetal bovine serum, 2 mM glutamine, and 100 μg/ml of an antibiotic–antimycotic cocktail from Sigma (penicillin, streptomycin, and gentamycin) at 37°C and 5% CO_2_ in a humidified incubator. Cells were grown to a density of 2–5 × 10^6^ cells/ml in 25 cm^2^ flat-bottom tissue culture flasks and passaged every third day.

### 2.6 *In vitro* model of latent tuberculosis

An *in vitro* nutritional starvation model of latent tuberculosis was developed according to the method described by Betts et al. (2002). Briefly, *M. tuberculosis* H_37_Rv was cultured and grown in nutrition-rich media, i.e., Soutan’s media containing 2% glycerol and 0.05% Tween 80, under constant shaking conditions at 200 rpm for 7 days. After 7 days, the culture was centrifuged at 5,000×*g* for 10–15 min, and the bacterial pellet was washed and transferred to PBS containing 0.05% Tween 80 (PBST) for 6 weeks under shaking conditions at 200 rpm in an incubator shaker. After 6 weeks of incubation, it was used as a latent TB culture and stored at −80°C until further use.

### 2.7 Infection of THP-1 derived macrophages with *M. tuberculosis* H_37_Rv

For infection experiments, 3 × 10^5^ THP-1 cells/well were plated in complete RPMI-1640 media containing 5% FBS and different glucose concentrations, i.e., 5.5 mM (normoglycemic condition), 15 mM, and 25 mM (hyperglycemic condition). D-mannitol was used as an osmotic control at the same molar concentrations. Approximately 20 ng/ml of phorbol myristate acetate (MP Biomedicals, USA) was added to the media for 24 h for differentiation of monocytes to macrophages. After 24 h, cell monolayers were then covered with fresh RPMI media containing no antibiotics and respective glucose concentrations, supplemented with 2% FBS, and incubated at 37°C and 5% CO_2_ for 24 h. After 48 h of cell plating, cells were divided into three groups, i.e., an uninfected group, an active TB group, and a latent TB group. Active *M. tuberculosis* culture was used to infect cells of the active TB group, whereas dormant *M. tuberculosis* culture was used to infect THP-1 cells of the latent TB group at a multiplicity of infection (MOI) of 1:5 (5 bacteria/1 cell) for 3 h (Iona et al., 2012). After 3 h, cells were washed twice with warm RPMI 1640 and replenished with complete RPMI 1640 medium containing different glucose concentrations and amikacin (50 μg/ml) to prevent extracellular replication of mycobacteria.

### 2.8 Colony forming units enumeration

Lungs and spleen were aseptically removed and homogenized in phosphate buffer saline (PBS) and plated on Middlebrook 7H11 agar (Becton-Dickinson, USA) containing 10% oleic acid-albumin-dextrose-catalase (OADC) (Difco-Becton Dickinson, USA). For the latent group, where BCG was given to animals prior to *M. tuberculosis* infection, 4 mg/ml of 2-thiophenecarboxylic acid hydrazide (TCH) (Sigma Aldrich) was added to 7H11 agar media to select for *M. tuberculosis* (Lecoeur et al., 1989). The latently and actively infected THP-1 cells grown under the above-mentioned glucose concentrations were lysed in 0.1% Triton X-100 for 5 min at different time points, i.e., 0 day, 1 day, 3 day, and 6 day of infection. Lysates were plated on Middlebrook 7H11 agar plates supplemented with 10% OADC in triplicate. Plates were incubated at 37°C in a 5% CO_2_ environment, and CFU counts were determined after 28 days of incubation.

### 2.9 Gene expression analysis of matrix metalloproteinases

The uninfected, latently, and actively infected THP-1 cells grown under different glucose concentrations were washed with FBS-free RPMI media and treated with a Trypsin-EDTA solution. After centrifugation at 1,600×*g* for 3 min, the cell pellet was resuspended in TRIzol. For gene expression studies in animals, lung tissue was directly transferred to 2–3 ml of TRIzol, depending on the size of the tissue. RNA was then isolated with the standard phenol–chloroform method (Adilakshmi et al., 2006). For quantitative real-time polymerase chain reaction (qRT-PCR), RNA (500 ng) was reverse transcribed using the Revert Aid First Strand cDNA Synthesis Kit (Thermo Scientific, USA). qRT-PCR amplification was done using SYBR^®^ Green chemistry on Roche LightCycler^®^ 96 Real-Time PCR Systems. The sequence of primers used for the amplification of different genes is given in **Table 1**.

**Table 1:** Primer sequence for various genes.

Gene	Primer Sequence		Annealing temperature
*16srRNA*	GTGGCGAACGGGTGAGTAAC ATGCATCCCGTGGTCCTATC	(Forward) (Reverse)	60°C
*hspX*	CACCACCCTTCCCGTTCAG TGGACCGGATCTGAATGTGC	(Forward) (Reverse)	56°C
*tgs1*	TCGTTAATGCTGCCCAACCT CCGAATTGTCTCTGTCCCCC	(Forward) (Reverse)	56°C
*tgs3*	GACATCACCTACCACGTCCG TACATCTCCCACAATGGCCG	(Forward) (Reverse)	64°C
*tgs5*	GATGGGCCAGAAGATGGACC CTTGGTAAACAGCAGCACGG	(Forward) (Reverse)	53°C
*mmp1* (Human)	ATGCACAGCTTTCCTCCACTG CAGCCCAAAGAATTCCTGCATT	(Forward) (Reverse)	57°C
*mmp2* (Human)	AACTACGATGATGACCGCAA CTCCTGAATGCCCTTGATGT	(Forward) (Reverse)	54°C
*mmp9* (Human)	GCCACTACTGTGCCTTTGAG AGAATCGCCAGTACTTCCCA	(Forward) (Reverse)	58°C
*β-actin* (Human)	AGAGCCTCGCCTTTGCCGATC CCCACCATCACGCCCTGGTGC	(Forward) (Reverse)	64°C
*mmp1* (Mouse)	ACTACAACTGACAACCCAAGAAAG AGGAGATGCCTAGAATCACAGT	(Forward) (Reverse)	62°C
*mmp2* (Mouse)	AACGGTCGGGAATACAGCAG AAACAAGGCTTCATGGGGGC	(Forward) (Reverse)	63°C
*mmp9* (Mouse)	CTCTCCTGGCTTTCGGCTG TCCGTGAGGTTGGAGGTTTTC	(Forward) (Reverse)	63°C
*gapdh* (Mouse)	AGCTTGTCATCAACGGGAAG TTTGATGTTAGTGGGGTCTCG	(Forward) (Reverse)	60°C

### 2.10 Cytokine determination

Cytokines were determined in THP-1 culture supernatants by using the Cytometric Bead Array (CBA) Human Soluble Protein Master Buffer Kit (Becton Dickinson, USA) and in mouse serum by using the CBA Mouse Th1/Th2/Th17 cytokine kit. In the serum and culture supernatants, protein inhibitor cocktail (Sigma Aldrich, USA) was added and stored at −80°C. The cytokine assay was performed according to the manufacturer’s protocol.

### 2.11 Matrix metalloproteinase determination

Matrix metalloproteinases were determined in the serum of mice using ELISA kits from ImmunoTag, USA. Protein inhibitor cocktail (PIC) (Sigma Aldrich, USA) was added to the serum and stored at −80°C. The assay was performed according to the manufacturer’s protocol.

### 2.12 Statistical analysis

The level of significance was determined by the Mann–Whitney test and the Kruskal–Wallis test using the GraphPad Prism package, version 8.0 (GraphPad Software, San Diego, USA) (Verma et al., 2021).

## 3 Results

### 3.1 Bacillary load in latent tuberculosis mice model

**Figure 1:**
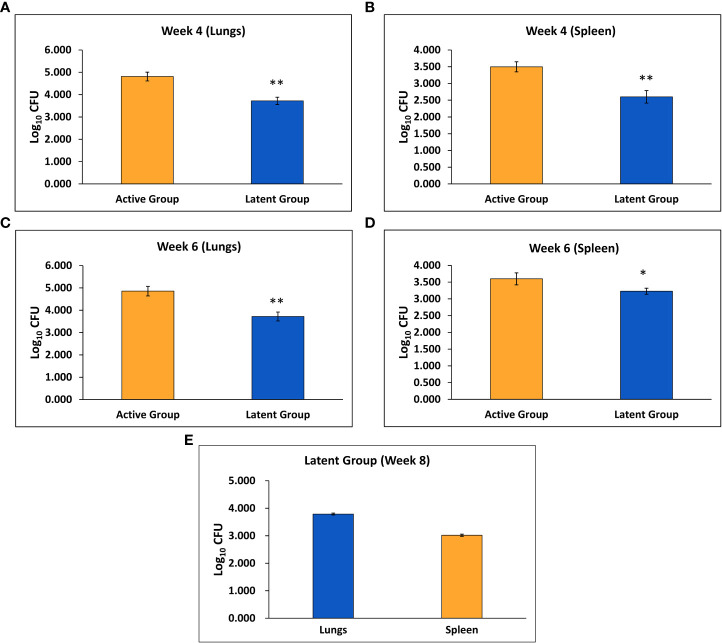
Log_10_ CFU counts of *M. tuberculosis* H_37_Rv in the active TB and latent TB groups. **(A)** After 4 weeks of infection in the lungs. **(B)** After 4 weeks of infection in the spleen. **(C)** After 6 weeks of infection in the lungs. **(D)** After 6 weeks of infection in the spleen. **(E)** After 8 weeks of infection in the lungs and spleen of the latent group. Values are Mean ± SE of five animals from each group. *p ≤0.05, **p ≤0.01 as compared to the active group.

Mice were infected with BCG through aerosol, and one day after immunization, the mean CFU counts of BCG in the lungs were found to be 1.92 ± 0.04 log_10_ (data not shown). After six weeks, the mean *M. tuberculosis* CFU counts after 1 day of infection were 1.84 ± 0.03 log_10_ (data not shown). Four weeks after challenge, the mean *M. tuberculosis* CFU counts in the lungs and spleen of the latent TB group were found to be significantly decreased as compared to the active TB group (**Figures 1A, B**). Similarly, after 6 weeks of infection, the mean CFU counts in the lungs and spleen were significantly lower in the latent TB group as compared to the active TB group (**Figures 1C, D**). Further, the mean CFU count of the lungs and spleen after 8 weeks of infection in the latent TB group were 3.79 ± 0.034 log_10_ and 3.02 ± 0.04 log_10_, respectively (**Figure 1E**). The CFU counts of the latent TB group after 4 and 6 weeks post-infection with *M. tuberculosis* confirmed the establishment of latent tuberculosis. The latent infection was sustained up to week 8 post-infection, as well, a stable CFU count was observed in the lungs and spleen of latent TB group animals.

### 3.2 Granuloma formation in the lungs of latent TB infected mice

**Figure 2:**
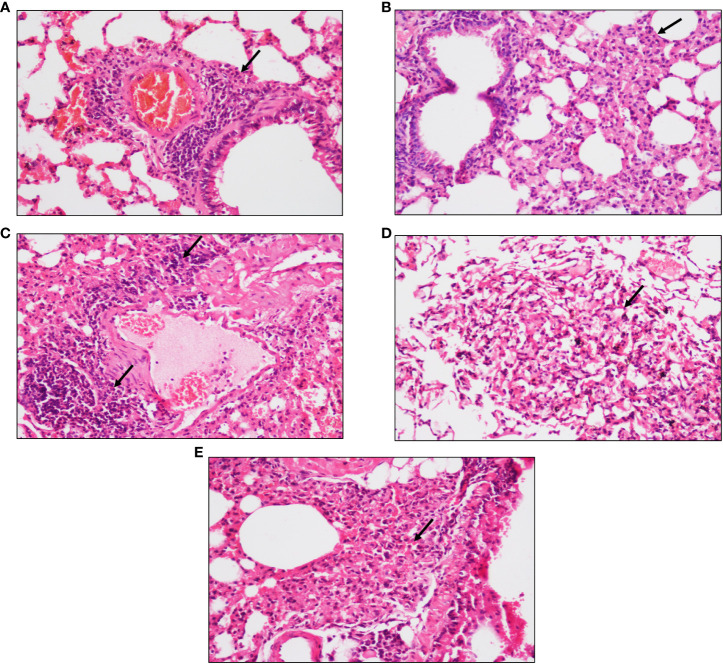
Representative images of hematoxylin and eosin staining of lung tissue of mice after 4, 6, and 8 weeks of infection with *M. tuberculosis* at ×20 magnification. **(A)** Section of the active TB group mice after 4 weeks. **(B)** Section of the latent TB group mice after 4 weeks. **(C)** Section of the active TB group mice after 6 weeks. **(D)** Section of the latent TB group mice after 6 weeks. **(E)** Section of the latent TB group mice after 8 weeks. Arrows indicate granulomas in the latent group and inflammation in the active group.

After 4 and 6 weeks of infection, peri-bronchial and peri-vascular inflammation was observed in the lungs of animals from the active TB group (**Figures 2A, C**) whereas in the latent TB group, the lungs were presented with parenchymal inflammation and inflammatory cell collection in air spaces. Also, histiocytic collection leading to the formation of sparse granulomas was observed after 4 weeks of infection in the latent TB group (**Figure 2B**). After 6 weeks of infection, collection of inflammatory cells at the pleural surface in the air spaces of the lungs was observed and ill-formed granulomas were observed in the latent TB group (**Figure 2D**). Lung tissues of animals from the latent TB group after 8 weeks of infection also displayed lymphoid and histiocytic collections, which indicated the formation of ill-formed granulomas (**Figure 2E**).

### 3.3 Expression of dormancy associated genes in lungs of latent TB model

**Figure 3:**
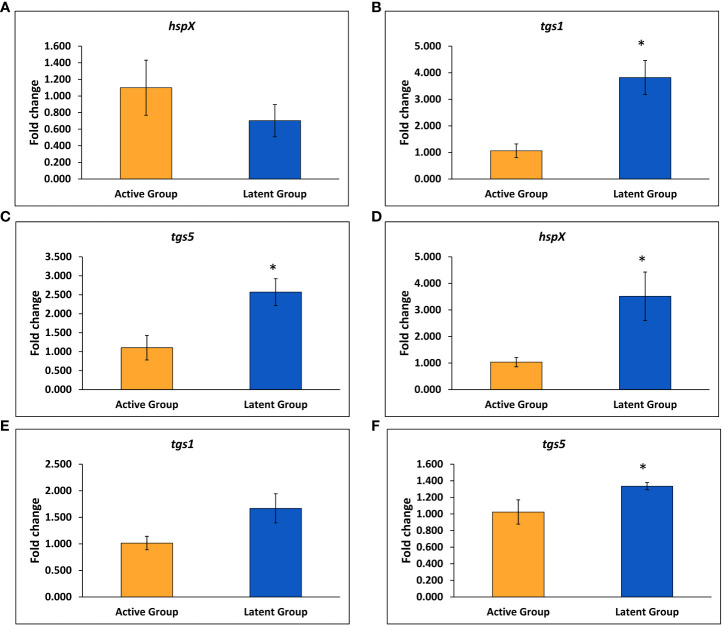
Expression of various genes in the latent TB group and active TB group. **(A)** *hspX* after 4 weeks. **(B)** *tgs1* after 4 weeks. **(C)** *tgs5* after 4 weeks. **(D)** *hspX* after 6 weeks. **(E)** *tgs1* after 6 weeks. **(F)** *tgs5* after 6 weeks. Values are Mean ± SE of three animals from each group. *p ≤0.05 as compared to the active TB and latent TB groups. 16srRNA was used as constitutive gene.

The expression of various latency-associated genes of *M. tuberculosis* was studied, including *hspX, tgs1, tgs3*, and *tgs5*. The gene expression of the *tgs1* and *tgs5* was found to be 3.8-fold and 2.6-fold upregulated, respectively, in the latent TB group as compared to the active TB group, and no significant change was observed in the expression of the *hspX* gene after 4 weeks of infection (**Figures 3A–C**). However, the expression of the *hspX* was found to be approximately 3.5-fold upregulated, and an approximately 1.4-fold increase in the expression of the *tgs5* was observed in the latent TB group as compared to the active TB group after 6 weeks of *M. tuberculosis* infection (**Figures 3D, F**), signifying the development of latency in the latent TB group. Also, the expression of *tgs1* was found to be increased non-significantly in latent TB group as compared to active TB group at week 6 (**Figure 3E**). No significant change was observed in the expression of *tgs3* after 4 and 6 weeks of infection between the latent and active TB groups (data not shown).

### 3.4 Development of diabetes in latently infected mice

**Figure 4:**
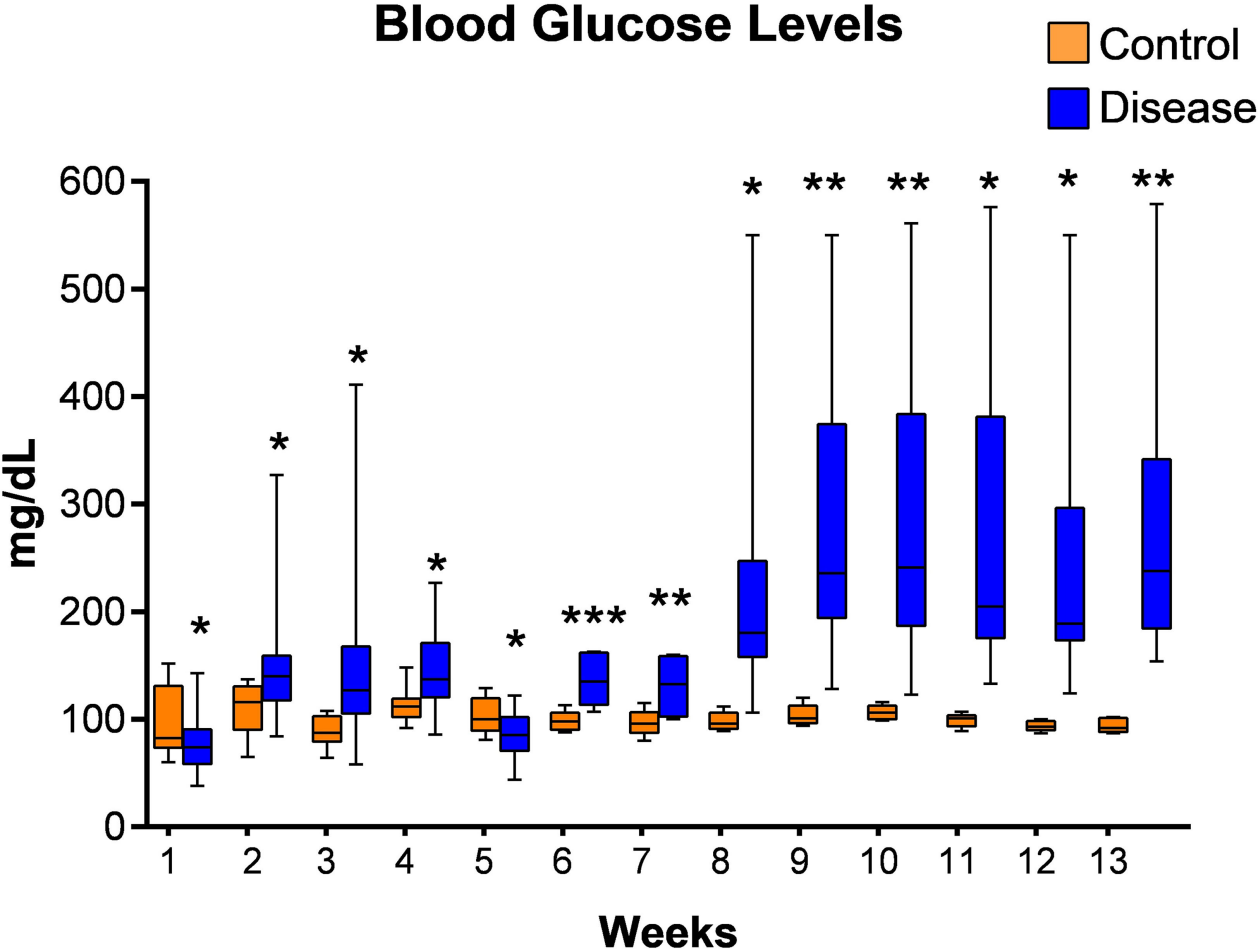
Blood glucose levels of mice from latent tuberculosis with diabetes group after streptozotocin treatment. Values are Mean ± SE of 24 animals until week 6, and 8–22 animals from week 7 until week 13. *p ≤0.05, **p ≤0.01, ***p ≤0.001 as compared to the control group. Blue bars indicate the streptozotocin treated group (latent tuberculosis with diabetes) and orange bars indicate the control group (healthy group without TB infection or diabetes).

Initially we tried to establish diabetes in latently infected mice with multiple low doses of streptozotocin and blood glucose levels were measured. However, diabetes in latently infected animals could not be established as blood glucose levels were found to be below 200 mg/dl until week 7 of their streptozotocin treatment at this dose (**Figure 4**). Thus, another single high dose of streptozotocin was given to these animals at week 7 of first streptozotocin treatment, and diabetes was found to have been successfully established in the latent TB group animals at week 8 as blood glucose levels were found to be greater than 200 mg/dl (**Figure 4**). Also, the pancreatic tissues of animals with latent TB and diabetes showed inflammation at weeks 9 (two weeks after diabetes development) and 13 (six weeks after diabetes development). The islets of Langerhans were densely inflamed, and loss of cellularity, necrosis, and admixture of cells were observed (**Supplementary Figures 1B, D**).

### 3.5 Expression analysis of matrix metalloproteinases

After the establishment of diabetes in latently infected mice, the animals were divided into three groups, i.e., Group-I (latent tuberculosis only), Group-II (latent tuberculosis with diabetes), and Group-III (latent tuberculosis with immunosuppression). Two weeks after the development of diabetes, i.e., week 9, the expression of the *mmp-1* was found to be significantly downregulated in Group-II as compared to Group-I, and no significant change in expression was observed in Group-III (**Supplementary Figure 2A**). Also, no significant change was observed in the gene expression of *mmp-2* and *mmp-9* in Groups-II and -III as compared to Group-I (data not shown). After six weeks of diabetes development, i.e., at week 13, an increase was observed in the expression of *mmp-1* in Group-II as compared to Group-I, but the increase was not significant (**Supplementary Figure 2B**). Moreover, no significant change was observed in the expression of *mmp-2* and *mmp-9* genes in Groups-I, -II, and -III respectively (data not shown). Thus, an increase in expression of *mmp-1* was observed in the diabetes group co-infected with latent tuberculosis, although the increase was non-significant. The levels of MMP-1, MMP-2, and MMP-9 were also measured in the serum of animals. At week 9, no significant change in the levels of MMP-1, MMP-2, and MMP-9 was observed between Groups-I, -II, and -III (data not shown). At week 13, the levels of MMP-1 were found to be significantly higher in Group-III in comparison to Groups-I and -II. The levels of MMP-1 and MMP-2 were significantly reduced in Group-II as compared to Group-I (**Supplementary Figures 2C, D**). The levels of MMP-9 were found to be the same between Group-I and Group-II. In Group-III, the levels of MMP-9 were significantly raised as compared to Group-II (**Supplementary Figure 2E**).

### 3.6 Cytokine levels in blood

At week 9, the levels of TNF-α were higher in Group-I, but the increase was not significant. The levels of IL-10, IFN-γ, IL-4, and IL-17 were found to be significantly increased in Group-III as compared to the control group, Group-I, and Group-II, but no change was found in the levels between Group-I and Group-II (data not shown). The levels of IL-6 were found to be significantly reduced in Group-II as compared to Group-I (**Supplementary Figure 3A**). No significant change was observed in the levels of IL-2 and TNF-α in any group (data not shown). At week 13, no significant change was observed in the levels of IL-10, TNF-α, IFN-γ, and IL-17 in any group (data not shown). A significant decrease was observed in the levels of IL-2 and IL-6 in Group-III as compared to the control group, Group-I, and Group-II (**Supplementary Figures 3B, C**). The levels of IL-4 were found to be significantly higher in Group-III as compared to Group-I, but no change was observed in the levels between the other groups (**Supplementary Figure 3D**).

### 3.7 High glucose conditions affects the macrophage bacterial clearing efficiency

**Figure 5:**
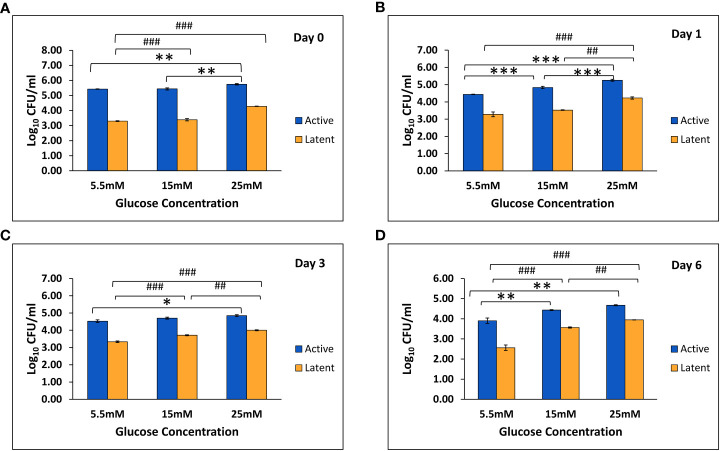
Log_10_ CFU counts of *M. tuberculosis* H_37_Rv in THP-1 cells infected with active and latent bacilli in the presence of different glucose concentrations at different time intervals. **(A)** On day 0 of infection. **(B)** On day 1 of infection. **(C)** On day 3 of infection. **(D)** On day 6 of infection. Values are Mean ±SE of three independent experiments. *p ≤0.05, **p ≤0.01, and ***p ≤0.001 represent the comparison between different glucose concentrations in the active group. ^##^p ≤0.01 and ^###^p ≤0.001 represent the comparison between different glucose concentrations in the latent group.

In THP-1 cells infected with active and latent cultures of *M. tuberculosis* H37Rv, the mean CFU counts were found to be significantly increased in the active as well as the latent group in the presence of 25 mM glucose concentration as compared to 5.5 mM and 15 mM glucose concentration on day 0 and day 1 (**Figures 5A, B**). On day 3, the mean CFU counts in the active group at 25 mM glucose were significantly increased as compared to 5.5 mM glucose, whereas in the latent group, the mean CFU counts were significantly increased in the presence of 15 mM and 25 mM glucose concentration as compared to 5.5 mM glucose concentration (**Figure 5C**). Similarly, on day 6 also the mean CFU counts in the active and latent groups were significantly increased with an increase in glucose concentration (**Figure 5D**). The CFU data indicated that as the glucose concentration is increased, the bacterial clearing capacity of macrophages is decreased in both active and latent infection.

### 3.8 *In vitro* expression analysis of matrix metalloproteinases

**Figure 6:**
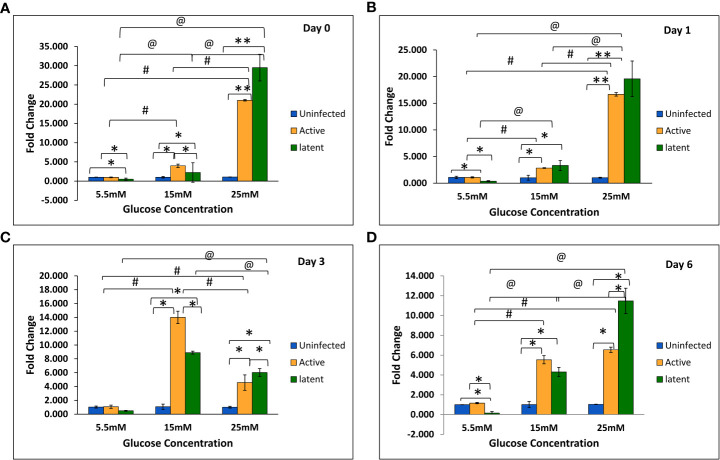
Expression of the *mmp-1* gene in the uninfected, active, and latent groups of THP-1 cells in the presence of different glucose concentrations at different time intervals. **(A)** On day 0 of infection. **(B)** On day 1 of infection. **(C)** On day 3 of infection. **(D)** On day 6 of infection. Values are Mean ±SE of three independent experiments. *p ≤0.05 represents the comparison between the uninfected, active, and latent groups within the same glucose concentration. **p ≤0.01 represents the comparison between the uninfected, active, and latent groups within the same glucose concentration. ^#^p ≤0.05 represents the comparison between different glucose concentrations in the active group. ^@^p ≤0.05 represents the comparison between different glucose concentrations in the latent group.

**Figure 7:**
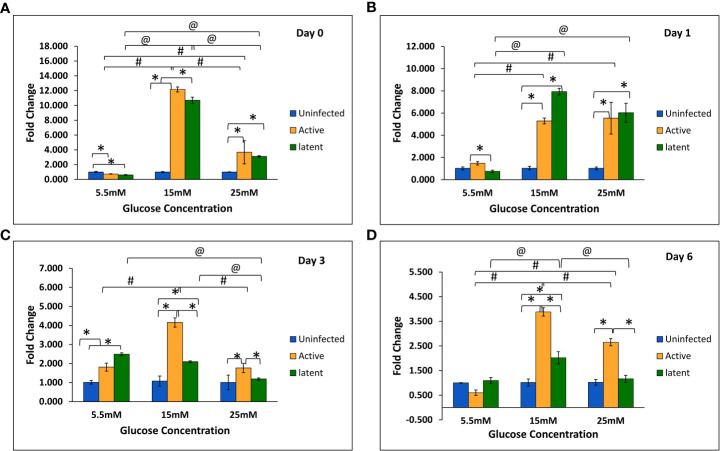
Expression of the *mmp-9* gene in the uninfected, active, and latent groups of THP-1 cells in the presence of different glucose concentrations at different time intervals. **(A)** On day 0 of infection. **(B)** On day 1 of infection. **(C)** On day 3 of infection. **(D)** On day 6 of infection. Values are Mean ±SE of three independent experiments. *p ≤0.05 represents the comparison between the uninfected, active, and latent groups within the same glucose concentration. ^#^p ≤0.05 represents the comparison between different glucose concentrations in the active group. ^@^p ≤0.05 represents the comparison between different glucose concentrations in the latent group.

**Figure 8:**
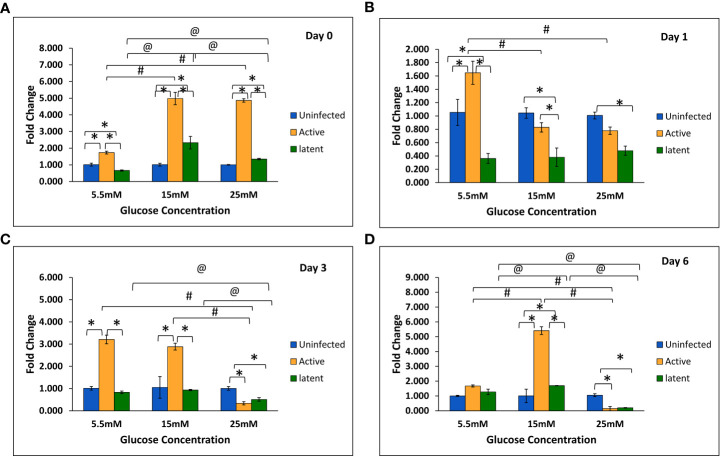
Expression of the *mmp-2* gene in the uninfected, active, and latent groups of THP-1 cells in the presence of different glucose concentrations at different time intervals. **(A)** On day 0 of infection. **(B)** On day 1 of infection. **(C)** On day 3 of infection. **(D)** On day 6 of infection. Values are Mean ±SE of three independent experiments. *p ≤0.05 represents the comparison between the uninfected, active, and latent groups within the same glucose concentration. ^#^p ≤0.05 represents the comparison between different glucose concentrations in the active group. ^@^p ≤0.05 represents the comparison between different glucose concentrations in the latent group.

With an increase in glucose concentration, the expression of the *mmp-1* gene was found to be upregulated in both active and latent groups as compared to the uninfected group at all time points, i.e., day 0, day 1, day 3, and day 6. At 25 mM glucose concentration, expression was observed to be 21-fold upregulated in the active group and approximately 29-fold upregulated in the latent group on day 0, whereas a 16.7-fold increase in the active group and a 19.7-fold increase in the latent group was observed on day 1 as compared to the uninfected group (**Figures 6A–D**). The expression of the *mmp-9* was found to be downregulated in the active and latent groups at 25 mM as compared to 15 mM glucose concentration at all time points, but the expression was significantly downregulated in the latent group as compared to the active group at later time points at high glucose conditions (**Figures 7A–D**). At higher glucose concentrations, the gene expression of *mmp-2* was found to be significantly increased in the active group at earlier time points, whereas at later time points it was observed to be downregulated. However, in the latent group a significant decrease in expression was observed on day 6 at 25 mM glucose concentration as compared to 5.5 mM and 15 mM glucose concentration (**Figures 8A–D**).

### 3.9 *In vitro* analysis of cytokines

The levels of different cytokines, i.e., IL-12, MCP-1, TNF-α, and MIP-1α, were determined in the THP-1 cell culture supernatants of the uninfected, active, and latent groups at different time intervals, i.e., 0 h, 6 h, 18 h, and 24 h of infection, in the presence of different glucose concentrations. With an increase in glucose concentration, the levels of MCP-1 were found to be decreased in the uninfected, active, and latent groups at all time points. This decrease in MCP-1 levels was more drastic in the active group as compared to the uninfected group with an increase in glucose concentration, which further explains reduced chemotaxis with an increase in glucose concentrations (**Supplementary Figures 4A–D**). The levels of TNF-α were found to be very high in both the active and latent groups with an increase in glucose concentrations (**Supplementary Figures 5A–D**). However, the levels of MIP-1α and IL-12 were found to be approximately similar in all the groups at 5.5 mM, 15 mM, and 25 mM glucose (data not shown).

## 4 Discussion

Diabetes is considered as a contributing factor to the dissemination of tuberculosis. Diabetes and active tuberculosis are well associated, yet the link between latent tuberculosis and diabetes has not been explored. Thus, a mice model of latent tuberculosis, developed by Zhang et al. (2009) was used and simultaneously diabetes was induced after establishment of latency which was confirmed by the CFU counts in lungs and spleen after infection with *M. tuberculosis*. The CFU counts were less than 10^4^ and remained stable, which affirmed the establishment of latent tuberculosis. The bacterial load in LTBI is presumed to be ≤10^4^ CFU, as the lower limit of detection for acid-fast smears is 10^4^ CFU/ml (Nuermberger et al., 2004; Zhang et al., 2009). Histological studies also supported the establishment of a latent tuberculosis model as granulomas were detected in the lungs after *M. tuberculosis* challenge. The results were supported by a study conducted by Dutta et al. (2014) wherein necrotic lung granulomas were observed after infection with *M. tuberculosis* in C3HeB/FeJ mice that were previously immunized with BCG. The development of granulomatous lesions after 2 and 10 weeks of low-aerosol infection with *M. tuberculosis* in the lungs of C57BL/6 mice has also been reported (Botha and Ryffel, 2002).

Alpha crystallin sustains the tubercle bacilli during the dormant or latent phase of infection, therefore qualifying HspX as a potential biomarker for latent TB infection (Mustafa et al., 1999; Botha and Ryffel, 2002). In our study, the gene expression of *hspX* was significantly upregulated in the latent TB group in comparison with the active TB group. This observation was in accordance with a study that revealed elevated HspX levels by tubercle bacilli in the latent state and its reversal to normal levels when exponential growth was resumed (Hu et al., 2006). It is known that *M. tuberculosis* can synthesize or accumulate triacylglycerol (TG), which leads to its long-term survival or persistence or dormancy under stress conditions (Daniel et al., 2004). *M. tuberculosis* synthesizes different TG synthases, which are responsible for the accumulation of TG under different stress conditions (Sirakova et al., 2006). The expression of the *tgs1* and *tgs5* genes has been observed to be upregulated in the latent TB group as compared to the active TB group. The results were supported by a study wherein the *tgs1* gene was found to be the prime contributor to TG synthesis in *M. tuberculosis* cultures grown under hypoxic and nitric oxide stress conditions (Daniel et al., 2004).

Further, diabetes was induced in latently infected mice using multiple, low doses of streptozotocin. A multiple, low-dose streptozotocin-induced diabetes model was developed by Leiter in 1982 using C57BL/6 mice (Leiter, 1982). After that, many researchers have also used this multiple, low-dose model to develop diabetes (McEvoy et al., 1984; Sun et al., 2005; Ventura-Sobrevilla et al., 2011). However, diabetes was not induced using multiple, low doses of streptozotocin in this study, which was thought to be due to BCG immunization as it has an important role in protection against diabetes. Various studies have observed that diabetes was not induced by multiple, low doses of streptozotocin in mice previously immunized with BCG (Baik et al., 1999; Rosa et al., 2013). Therefore, another single high dose of streptozotocin was given to the animals. and diabetes was successfully induced in latently infected mice.

To understand the crosstalk between intracellular mycobacteria and host macrophages, the THP-1 monocytic cell line was grown under hyperglycemic conditions and infected with latent as well as active cultures of *M. tuberculosis* for 3 h. Accordingly, mycobacterial phagocytosis by macrophages under high glucose conditions was studied. THP-1 cells were used as model phagocytic cells (Ranaivomanana et al., 2018; Riaz et al., 2020). CFU enumeration at normal glucose conditions suggested that the dormant bacilli were better contained within the macrophages as compared to the active bacilli. The data was supported by a study wherein the growth of dormant bacilli was better suppressed by macrophages as compared to aerobic bacilli (Iona et al., 2012). However, as the glucose concentration increased, the CFU counts in both the active and latent TB groups increased significantly as compared to normal glucose conditions at each time point. Similar results were obtained in a study in which bacterial internalization and killing were reduced by macrophages isolated from diabetic mice (Alim et al., 2017). Also, Gomez et al. observed that the monocytes isolated from diabetic patients had reduced intake of *M. tuberculosis* in comparison to monocytes isolated from nondiabetic patients (Gomez et al., 2013).

Cytokines play a critical role in active and latent TB infection. Th1 cytokines differentiate between latent tuberculosis and active tuberculosis, as their levels are higher in LTBI (Wu et al., 2017). A study by Kumar et al. found that latent tuberculosis infection and diabetes co-pathology are characterized by decreased systemic Th1 cytokine levels (Kumar et al., 2014). In this study, the levels of TNF-α were found to be lower in the latent group as compared to the active group at 0 h. However, with an increase in time, the levels of TNF-α were found to be higher in the latent group as compared to the active group at high glucose conditions. In a mouse model of latent tuberculosis, TNF-α levels were found to be decreased non-significantly in the latent tuberculosis with diabetes group at week 9 of diabetes induction as compared to latent tuberculosis alone; however, levels were comparable between both groups at week 13. The results were in contrast to a study in which reduced levels of type-1 and type-17 cytokines along with diminished circulating levels of pro-inflammatory cytokines were reported in individuals with latent tuberculosis and diabetes as compared to individuals with latent tuberculosis without diabetes (Kumar and Babu, 2017). The possible reasons for this variation could be the species difference as the present study was performed on mice, and another possible reason could be the early time points as the sacrifice was done after the second (week 9) and sixth (week 13) weeks of diabetes establishment. Th2 cytokines, i.e., IL-4, IL-6, IL-10, IL-13, etc., inhibit Th1 responses and thus cross-regulate and influence the progression to active tuberculosis (O’Garra et al., 2013). In the mouse model, the levels of IL-6 and IL-10 were found to be significantly decreased in the latent tuberculosis with diabetes group in comparison to the latent tuberculosis only group. The results were supported by a study in which no difference in the levels of IL-4, IL-5, IL-6, and IL-13 and a decrease in IL-10 levels were reported between latently infected individuals with diabetes and those without diabetes (Kumar et al., 2014).

Along with the cytokines, levels of chemokines, i.e., monocyte chemotactic protein-1 (MCP-1) and macrophage inflammatory protein-1a (MIP-1α), were measured. MCP-1 plays an important role in latent infection, primarily in the establishment and maintenance of granulomas by recruiting leukocytes at the site of infection (Deshmane et al., 2009). In this study, the levels of MCP-1 were found to be decreased in the culture supernatants of the active group as compared to the latent and uninfected groups, and the levels were greatly reduced at higher glucose concentrations as compared to normal glucose concentrations in both the active and latent groups. Results were supported by a study in diabetic mice infected with *M. tuberculosis*, which showed reduced levels of MCP-1 in lung lysates, resulting in delayed migration of dendritic cells from the lungs to lymph nodes (Vallerskog et al., 2010). Chemokines like MIP-1α, CCL4, and CCL5 function in synergy with IFN-γ as pro-inflammatory chemokines (Dorner et al., 2002). However, no change in the levels of MIP-1α in the culture supernatants was observed between the uninfected, active, and latent groups or between normal glucose and high glucose conditions. The results were supported by a study in which no difference was observed in the secretion and mRNA expression of CCL3 upon *M. tuberculosis* infection between extrapulmonary and pulmonary TB patients (Hasan et al., 2009).

ECM plays a vital role in the structural composition of granulomas in terms of leucocyte trafficking in and out of this dynamic environment (Gonzalez-Juarrero et al., 2001). Interestingly, MMPs appear to play a key role in both granuloma formation and in lung tissue destruction. In the present study, the expression of the *mmp-1* gene was observed to be increased non-significantly at week 13 in latently infected mice with diabetes in comparison with non-diabetic mice. Similarly, in *in vitro* studies, the gene expression of *mmp-1* was observed to be significantly upregulated in the latent group as compared to the active group at high glucose concentrations. The results were supported by a study wherein the circulating levels of MMP-1 were found to be elevated in active pulmonary TB patients as compared to individuals with LTBI (Andrade et al., 2015). However, no significant change was observed in the protein levels of MMP-1 at week 9, and a significant decrease was observed in levels at week 13 in the serum of latently infected mice with diabetes compared to those without diabetes. Results were comparable to a study in which no significant difference was observed in the plasma levels of MMP-1 between tuberculosis patients with or without diabetes (Andrade et al., 2014). The MMP-9 elevation was related with monocyte and macrophage recruitment, which is essential for the maturation of granulomas (Volkman et al., 2010). In this study, the gene expression of *mmp-9* was found to be significantly decreased in the latent group as compared to the active group at high glucose concentrations in THP-1 cells. The results were in accordance with a study on HIV-TB in which the concentrations of MMP-1, -2, -3, and -9 were decreased in the sputum of TB patients with HIV as compared to those without HIV (Walker et al., 2017). However, *in vivo* studies did not show any significant difference either in gene expression or in the serum levels of MMP-9 in actively infected mice or in latently infected mice with or without diabetes. The results were supported by another study in which no changes in the circulating levels of MMP-9 were observed between TB patients with or without diabetes (Kumar et al., 2018).

In conclusion, an animal model was employed to study latent tuberculosis and diabetes. Although animal models of tuberculosis and diabetes co-morbidity have been developed in the past, no animal model of latent tuberculosis and diabetes has been developed so far. This model of latent tuberculosis and diabetes was further used to study the role of diabetes in the activation of latent tuberculosis (Verma et al., 2021). Further, diabetes and hyperglycemic conditions lead to a decrease in levels of MCP-1, increased gene expression of *mmp-1*, and decreased gene expression of *mmp-9*, which together may lead to a disruption in the process of granuloma formation and in the activation of latent TB infection.

## Data availability statement

The original contributions presented in the study are included in the article/**Supplementary Material**. Further inquiries can be directed to the corresponding author.

## Ethics statement

The animal study was reviewed and approved by the Institutional Animal Ethical Committee, PGIMER, Chandigarh with ref. no. 89/90/IAEC/616 and the Animal Ethical Committee, ICGEB, New Delhi with ref. no. ICGEB/IAEC/02042019/TACF-PGIMER-16.

## Author contributions

AV—Conception & design of the work, acquisition, analysis, and interpretation of data, wrote the main manuscript text and prepared figures. MK, PL, LS—Acquisition of data. DA, IV, BR, SB—Interpretation of data. SS—Conception and design of the work, interpretation of data and substantively revised the work. All authors listed have made a substantial, direct, and intellectual contribution to the work and approved it for publication.
