# Supplementary material for: Immunological aspects of host–pathogen crosstalk in the co-pathogenesis of diabetes and latent tuberculosis
Source: Front Cell Infect Microbiol. 2023 Jan 26;12:957512. doi: 10.3389/fcimb.2022.957512 (PMC9909355; doi:10.3389/fcimb.2022.957512)
Supplement: Supplementary file 1 [file DataSheet_1.docx]

**Supplementary Figures**

B

A


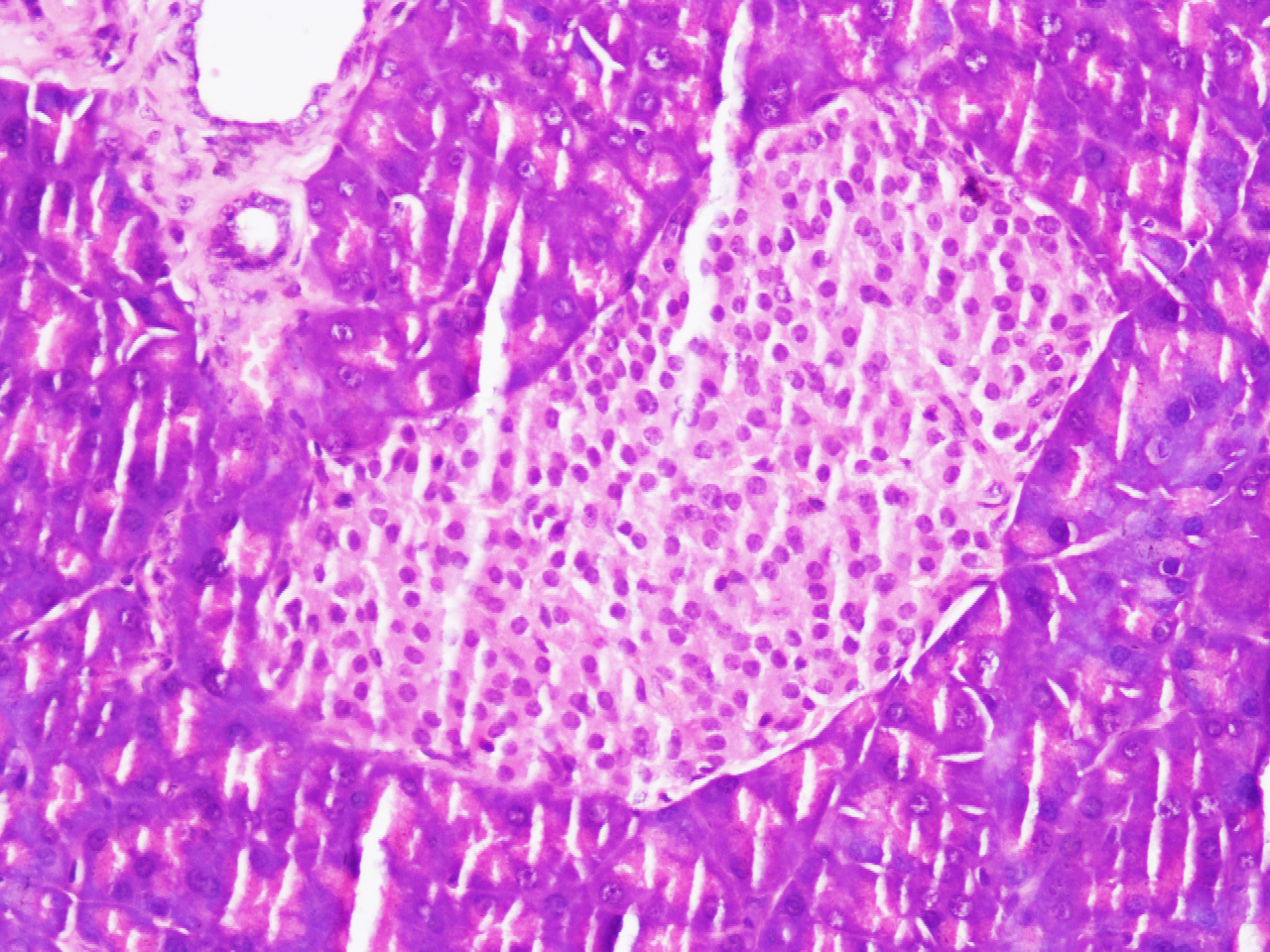

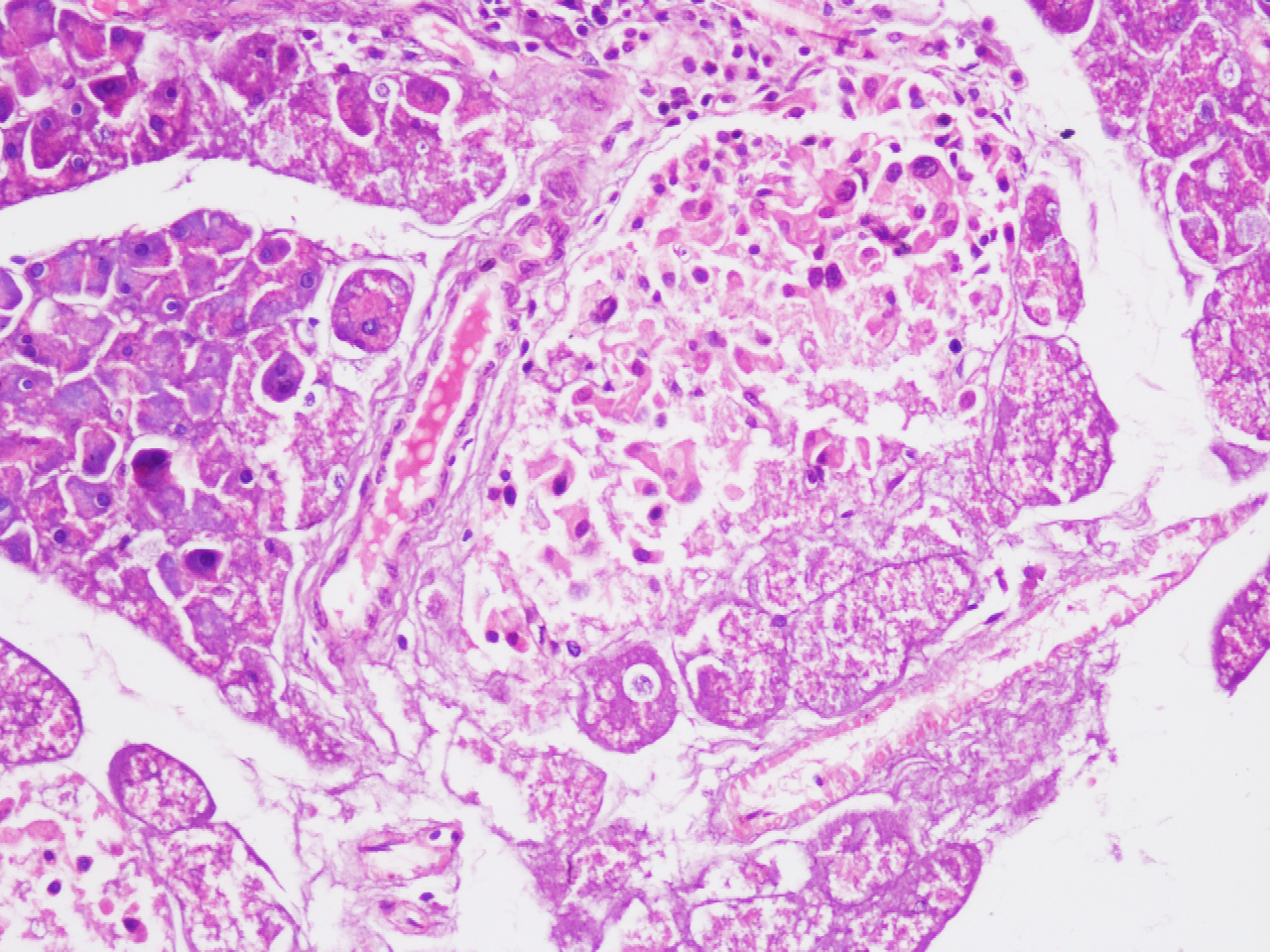


D

CA


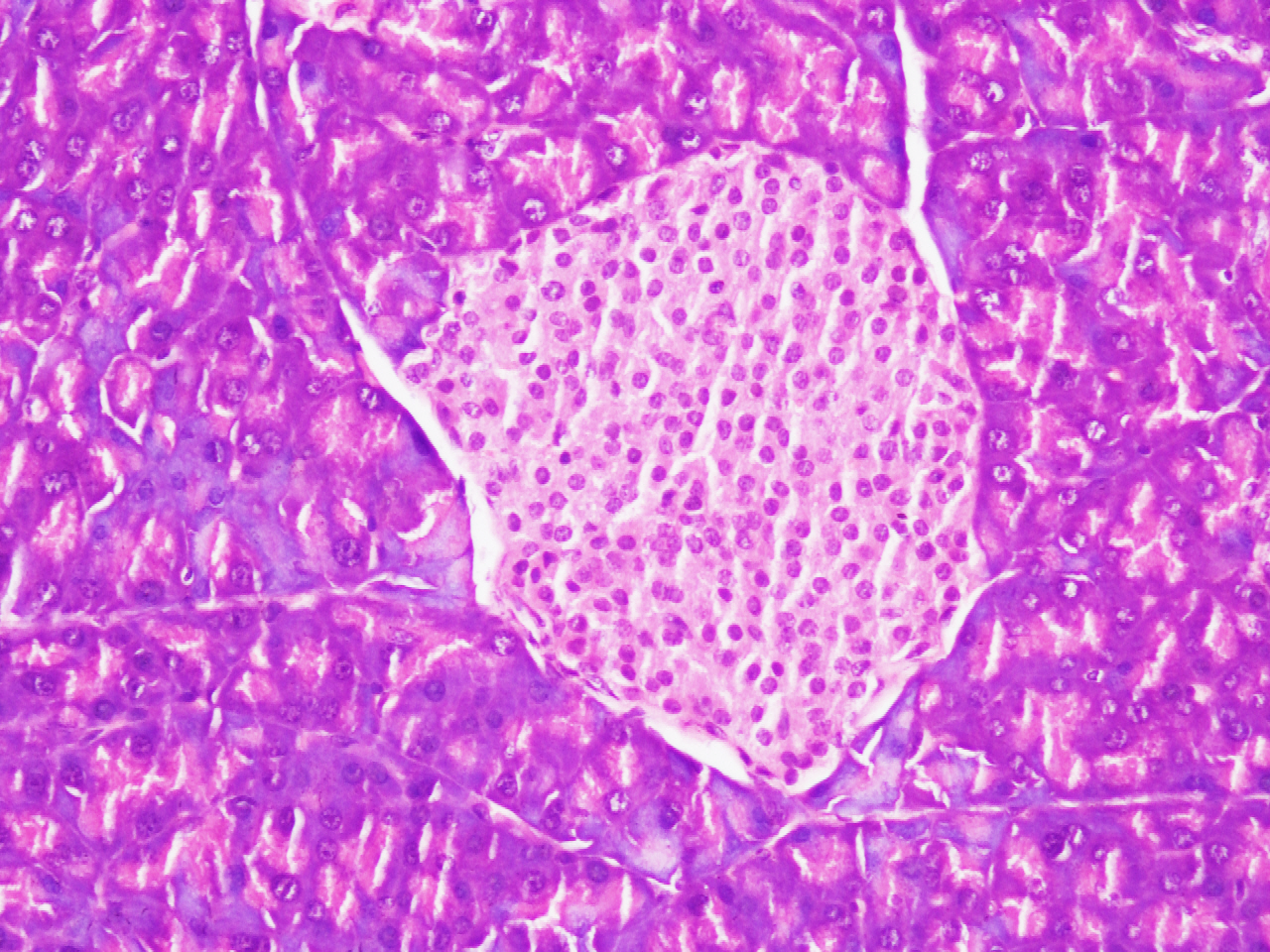

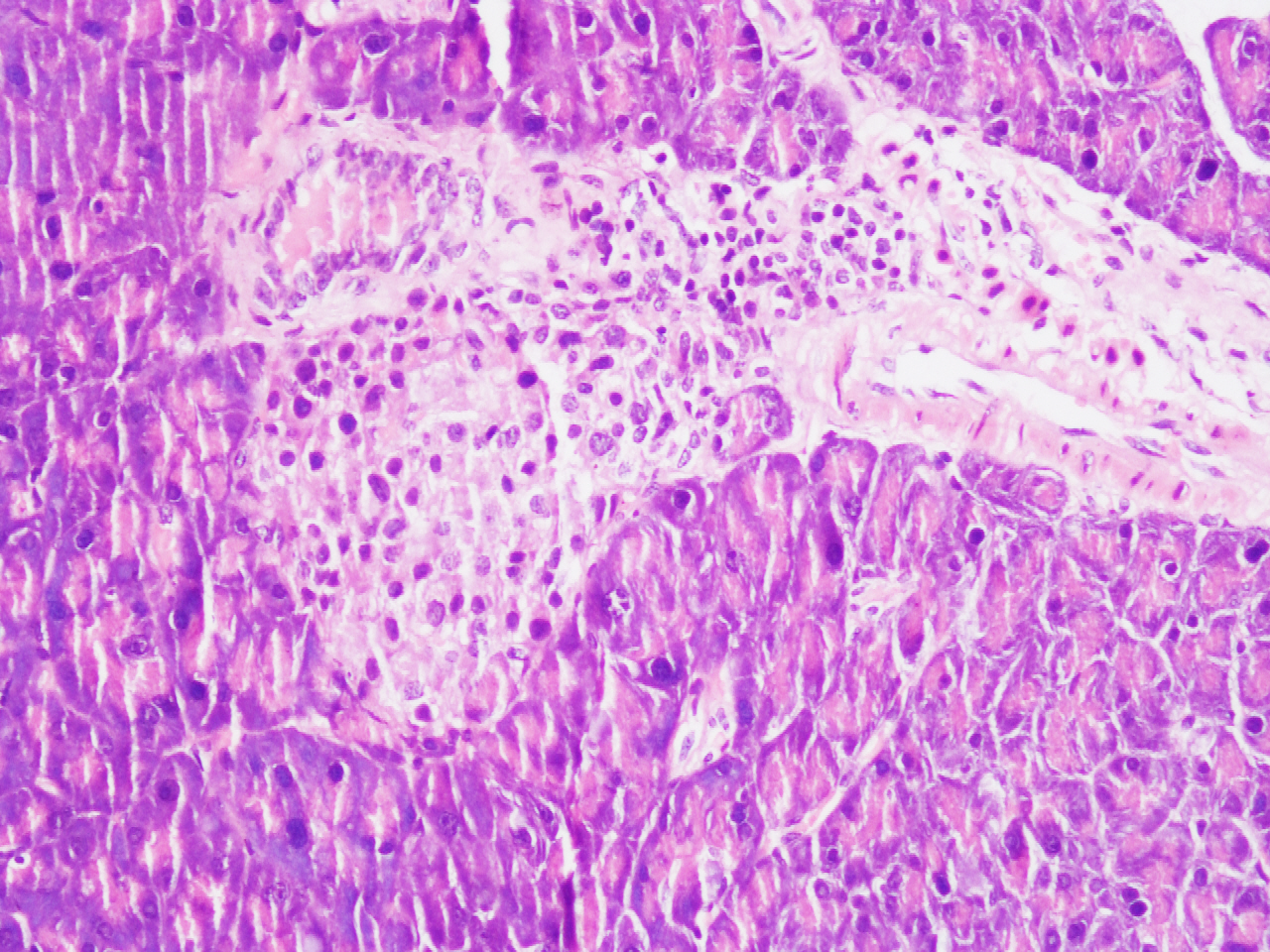


**Figure 1. Representative images of Hematoxylin & Eosin staining of pancreatic tissue of mice from control group and latent tuberculosis with diabetes group after 9 and 13 weeks of streptozotocin treatment.** A) Section of control group mice at week 9 at 20X magnification B) Section of latent TB with diabetes group mice at week 9 at 20X magnification C) Section of control group mice at week 13 at 20X magnification D) Section of latent TB with diabetes group mice at week 13 at 20X magnification. Arrows indicate the pancreatic islets. Control group - Healthy group without TB infection or diabetes.

B

CA

D

A

E

**Figure 2.** **Gene expression and serum levels of various matrix metalloproteinases of mice after 9 and 13 weeks of streptozotocin treatment.**A) Expression of *mmp-1* at week 9 in lungs. B) Expression of *mmp-1* at week 13 in lungs. C) Serum levels of MMP-1 at week 13. D) Serum levels of MMP-2 at week 13. E) Serum levels of MMP-9 at week 13. Values are Mean±SE of 3 animals. *p≤0.05 represents the comparison between Control vs Group-I, Control vs Group-II and Control vs Group-III #p≤0.05 represents the comparison between Group-I vs Group-II and Group-I vs Group-III @p≤0.05 represents the comparison between Group-II and Group-III. Control group – Healthy animals without tuberculosis or diabetes, Group-I – latent tuberculosis only, Group-II – latent tuberculosis with diabetes and Group-III – latent tuberculosis with immunosuppression.

A

B

CA

D

**Figure 3. Serum levels of different cytokines in animals from Control group, latent tuberculosis only, latent tuberculosis with diabetes and latent tuberculosis with immunosuppression group after 9 and 13 weeks of diabetes induction** A) IL-6 after 9 weeks. B) IL-2 after 13 weeks C) IL-6 after 13 weeks
D) IL-4 after 13 weeks. Values are Mean≤SE of 5 animals. *p≤0.05 as compared to Control group, #p≤0.05 as compared to Group-I and @p≤0.05 as compared to Group-II. Control group – Healthy animals without tuberculosis or diabetes, Group-I – latent tuberculosis only, Group-II – latent tuberculosis with diabetes and Group-III – latent tuberculosis with immunosuppression.


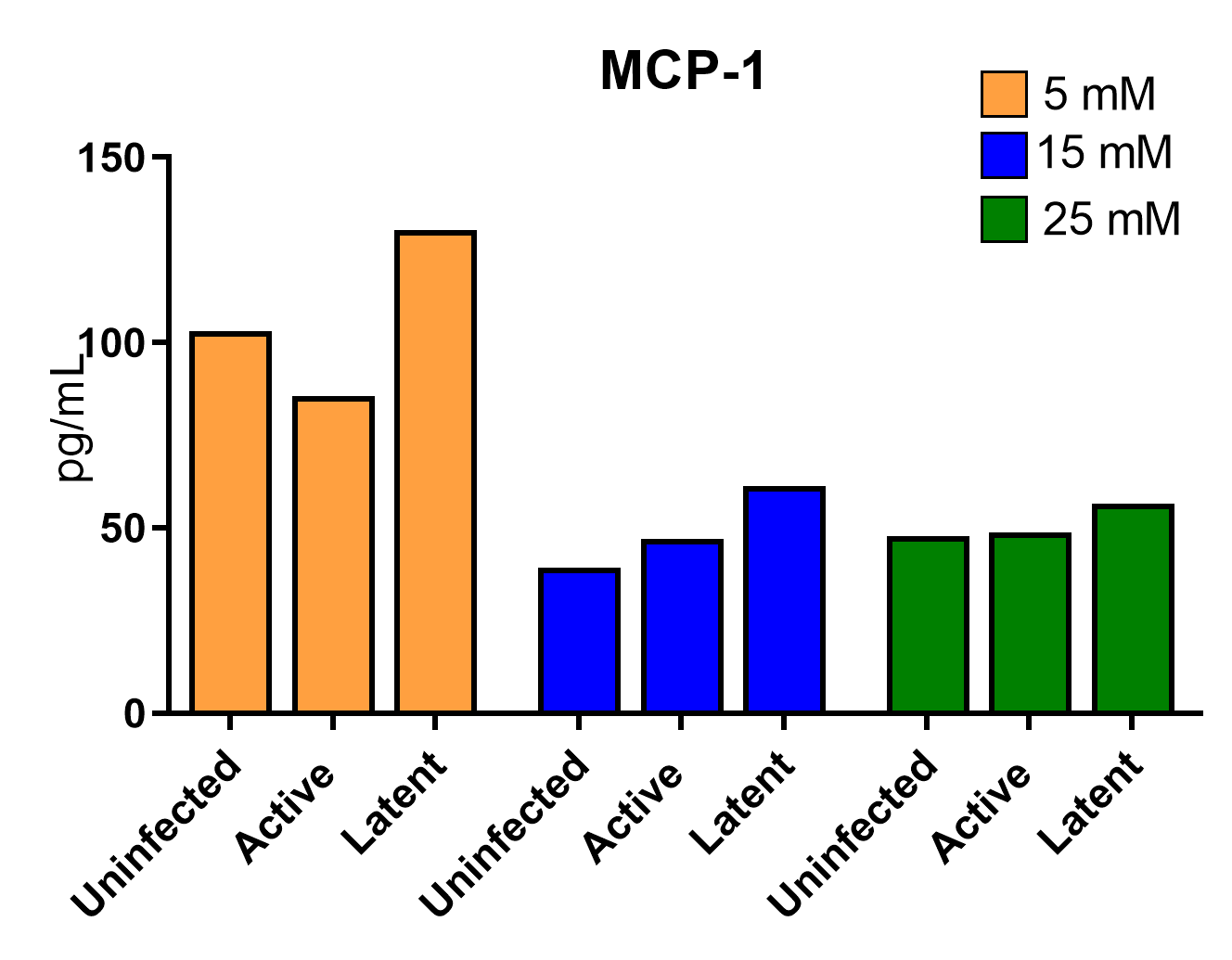

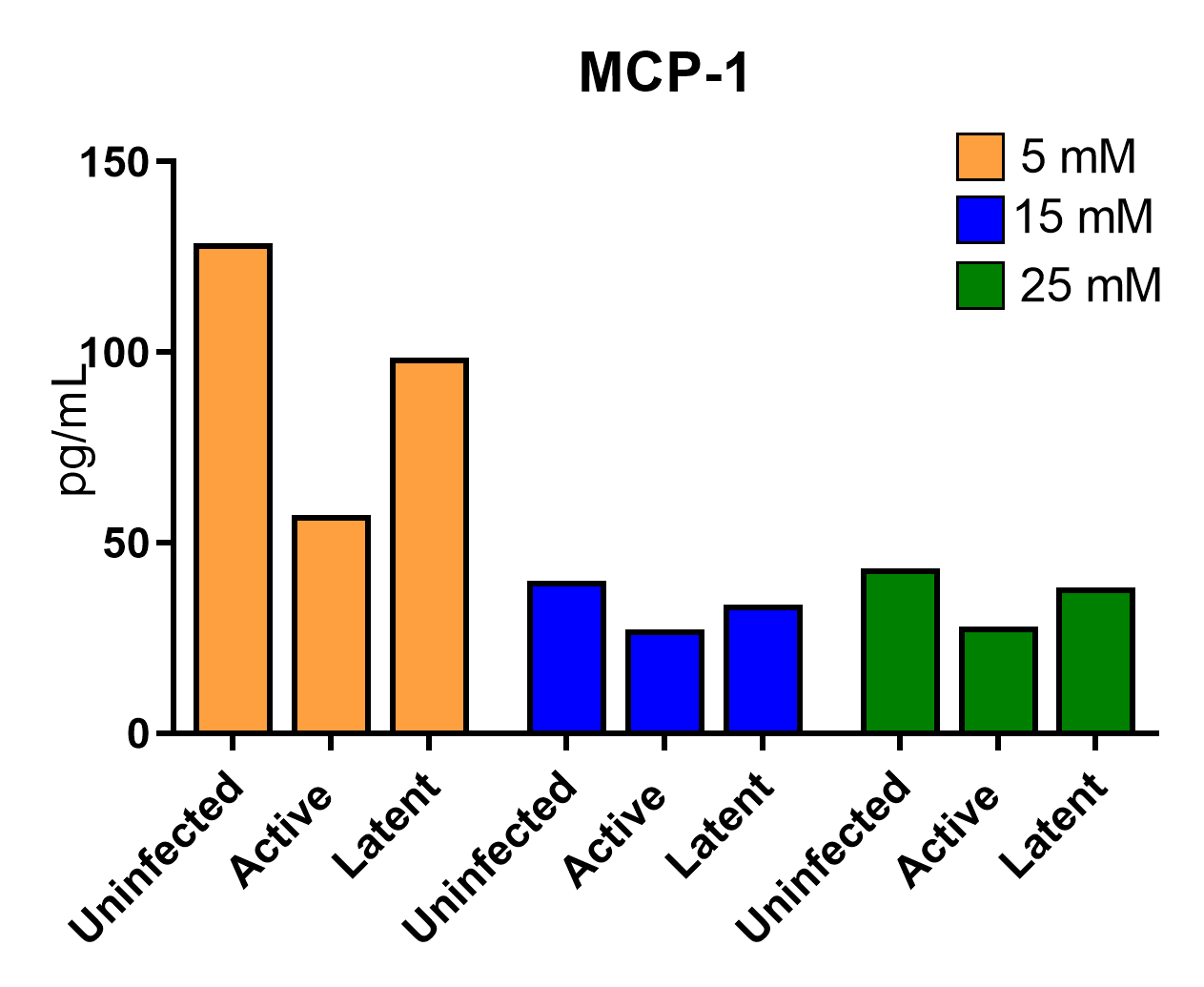

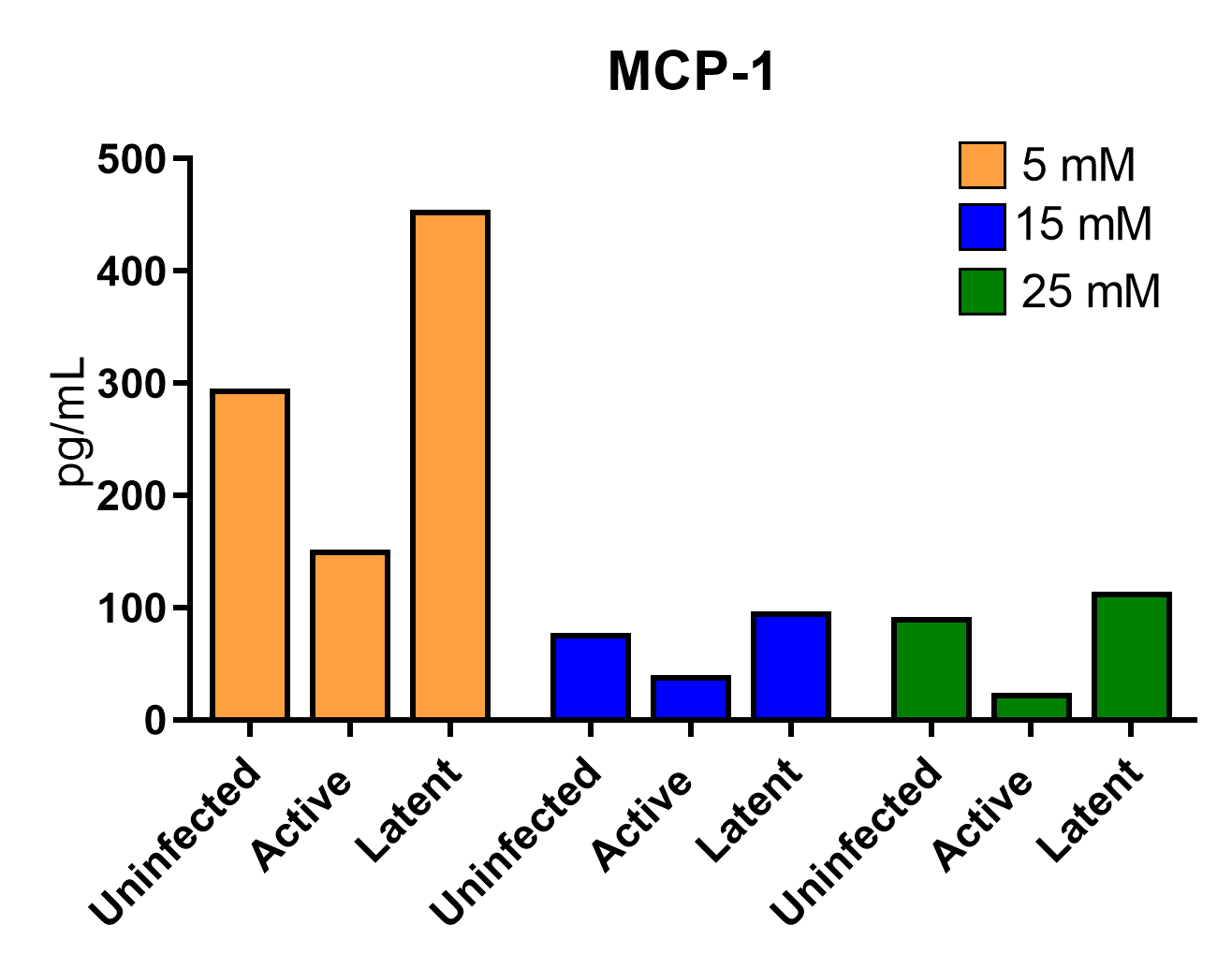

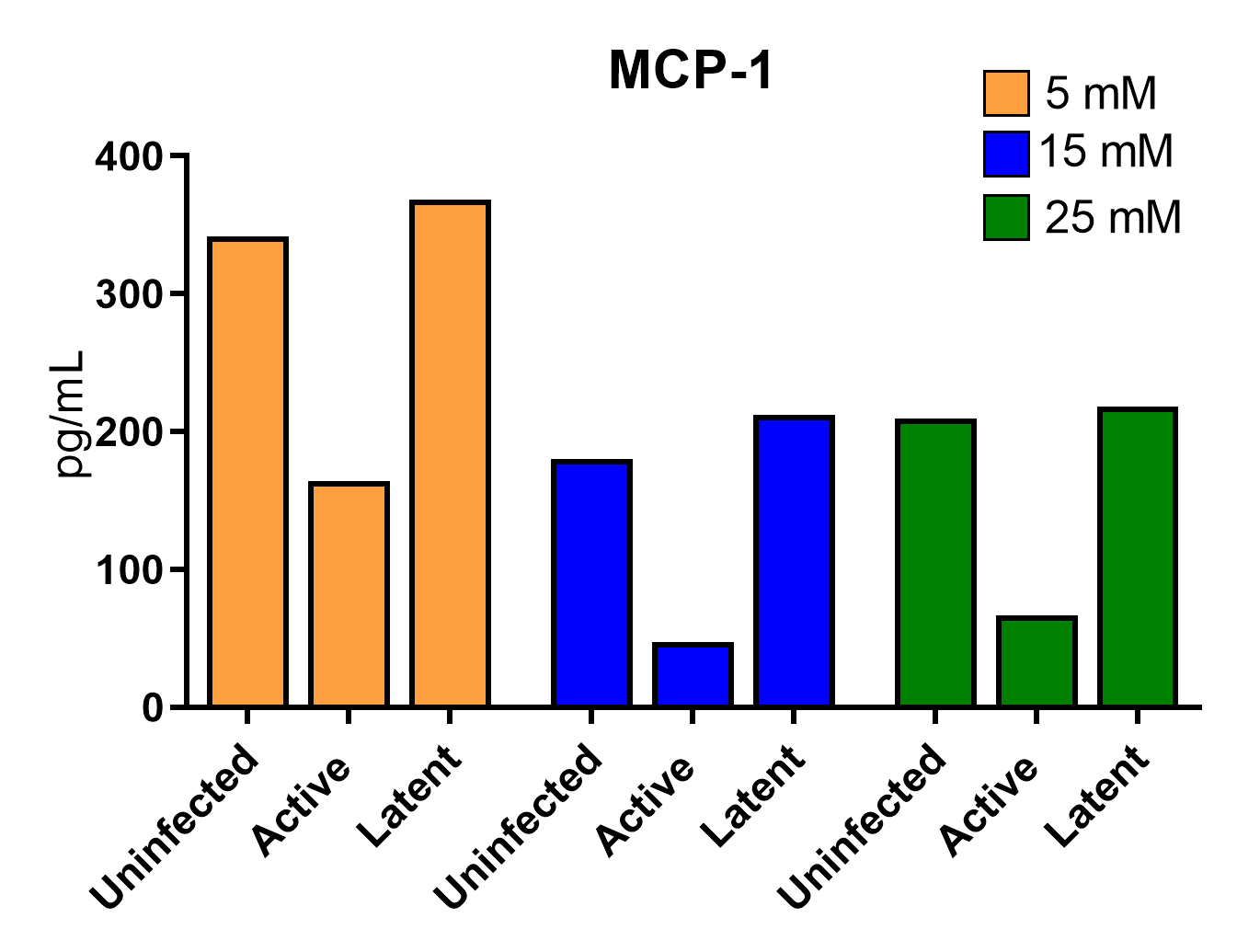


CA

D

A

B

**Figure 4. Levels of monocyte chemotactic protein 1 in the culture supernatants of different groups of THP-1 cells i.e. uninfected, active and latent group in presence of different glucose concentrations at different time intervals.** A) At 0h of infection. B) At 6h of infection. C) At 18h of infection. D) At 24h of infection. Experiments were conducted twice and data is represented as mean values.


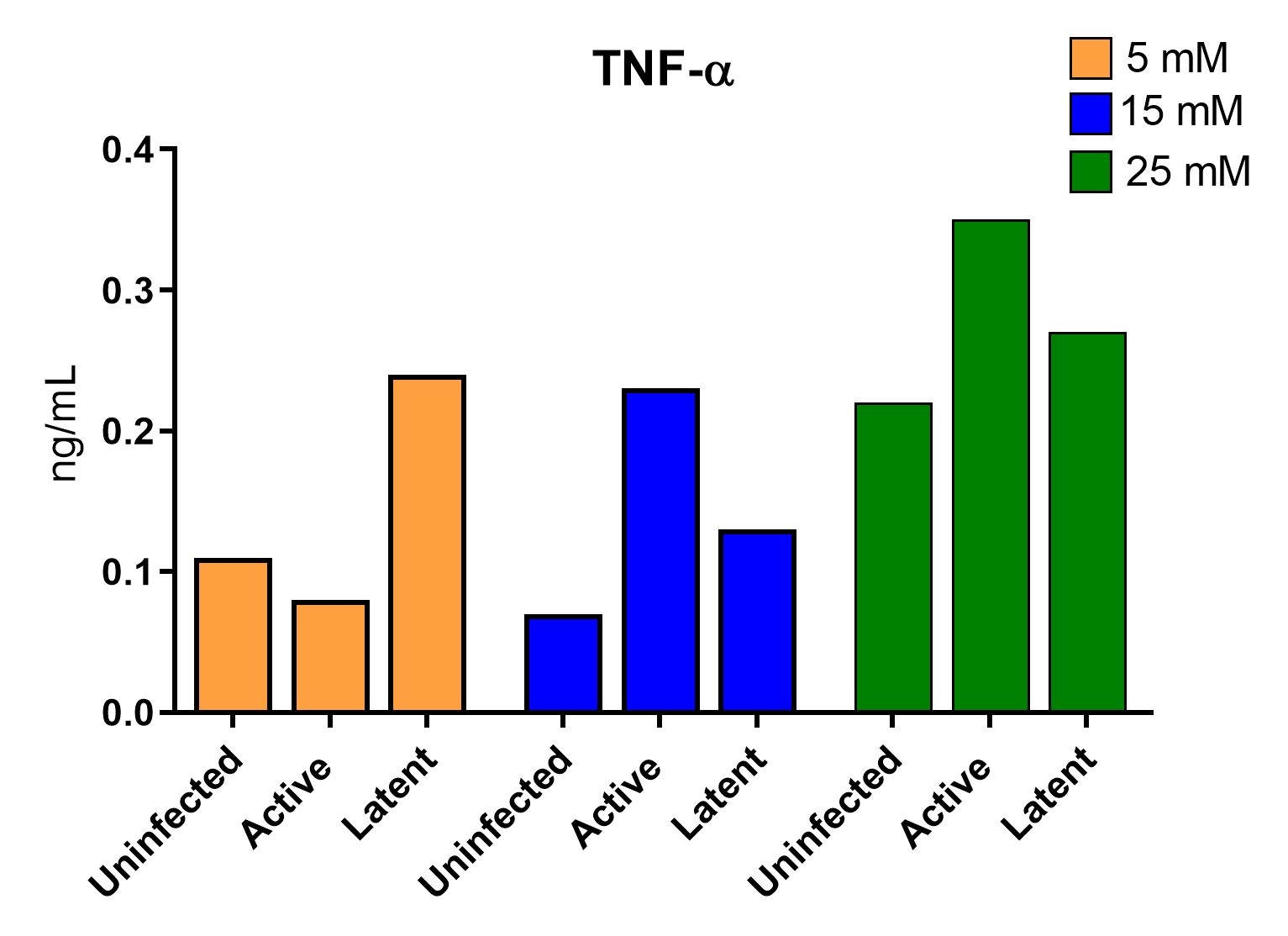

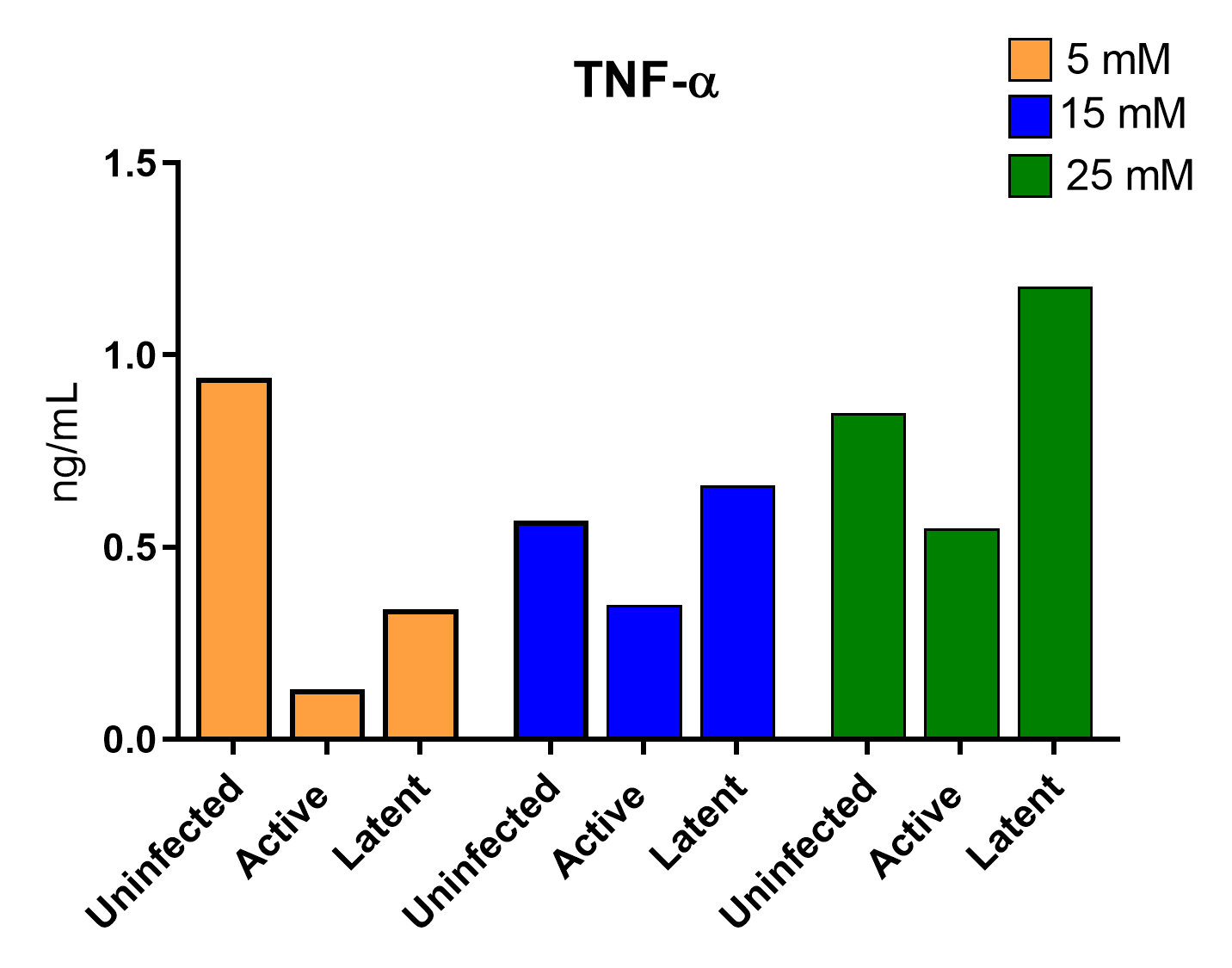

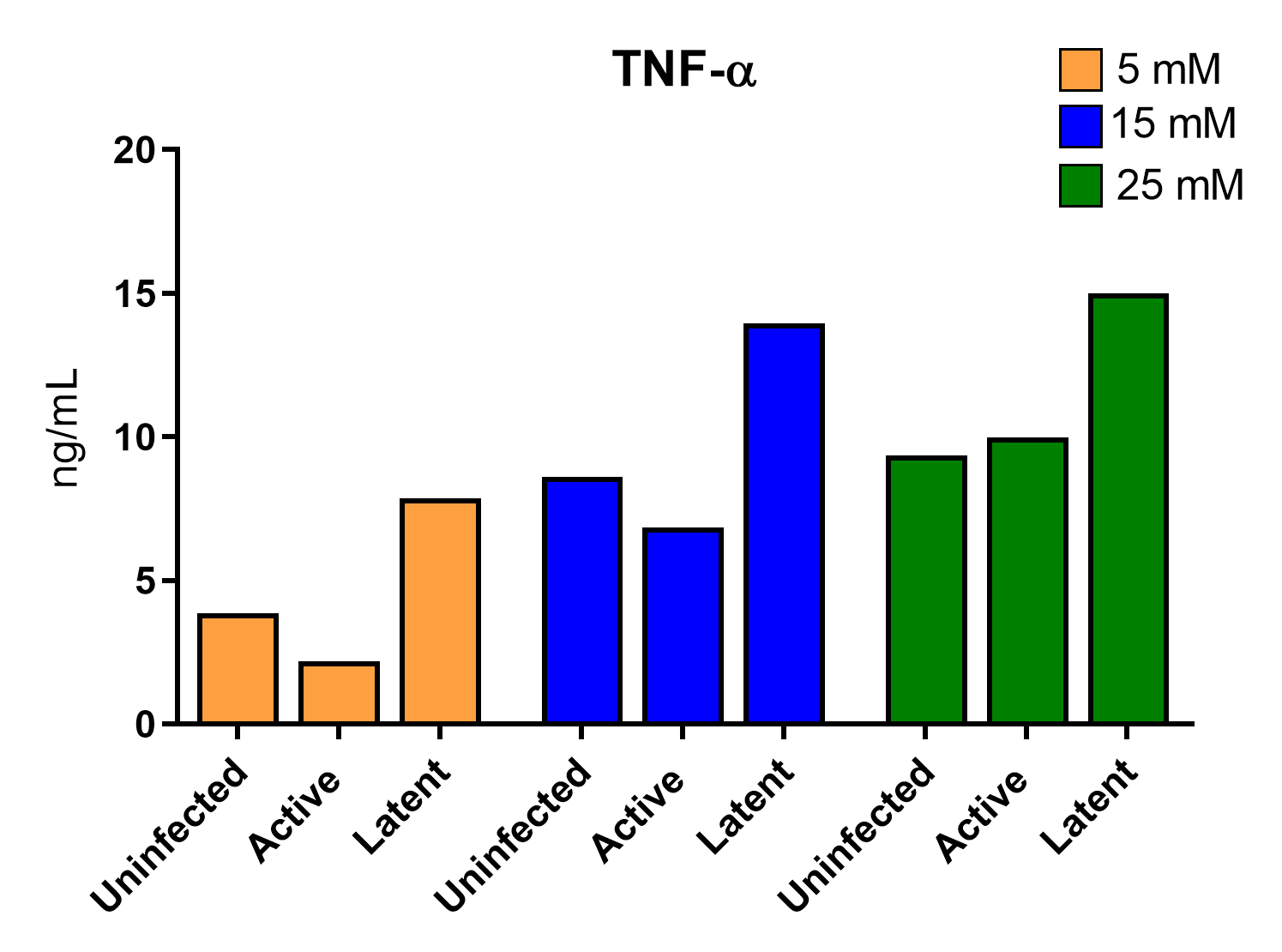

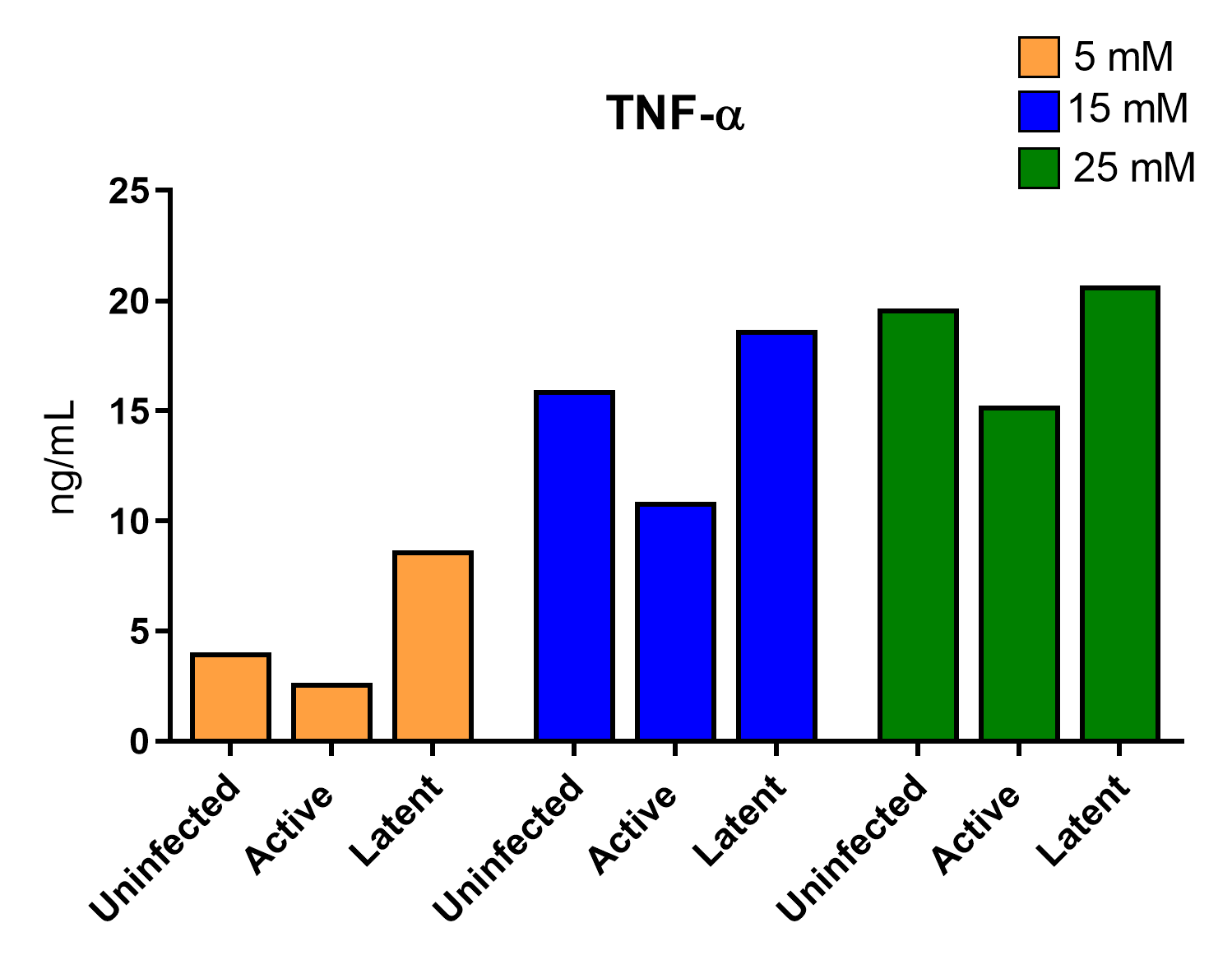


CA

D

A

B

**Figure 5. Levels of tumour necrosis factor-α in the culture supernatants of different groups of THP-1 cells i.e. uninfected, active and latent group in presence of different glucose concentrations at different time intervals.** A) At 0h of infection. B) At 6h of infection. C) At 18h of infection. D) At 24h of infection. Experiments were conducted twice and data is represented as mean values.
